# Chronic Exposure to Palmitate Impairs Insulin Signaling in an Intestinal L-cell Line: A Possible Shift from GLP-1 to Glucagon Production

**DOI:** 10.3390/ijms19123791

**Published:** 2018-11-28

**Authors:** Agnese Filippello, Francesca Urbano, Stefania Di Mauro, Alessandra Scamporrino, Antonino Di Pino, Roberto Scicali, Agata Maria Rabuazzo, Francesco Purrello, Salvatore Piro

**Affiliations:** Department of Clinical and Experimental Medicine, Internal Medicine, Garibaldi-Nesima Hospital, University of Catania, 95122 Catania, Italy; agnese.filippello@gmail.com (A.F.); francescaurbano@hotmail.it (F.U.); 8stefaniadimauro6@gmail.com (S.D.M.); alessandraska@hotmail.com (A.S.); antonino.dipino@unict.it (A.D.P.); robertoscicali@gmail.com (R.S.); rabuazzo@unict.it (A.M.R.); spiro@unict.it (S.P.)

**Keywords:** intestinal L-cells, lipotoxicity, insulin resistance, proglucagon

## Abstract

Obesity and type 2 diabetes mellitus (T2DM) are characterized by insulin resistance and impaired glucagon-like peptide-1 (GLP-1) secretion/function. Lipotoxicity, a chronic elevation of free fatty acids in the blood, could affect insulin-signaling in many peripheral tissues. To date, the effects of lipotoxicity on the insulin receptor and insulin resistance in the intestinal L-cells need to be elucidated. Moreover, recent observations indicate that L-cells may be able to process not only GLP-1 but also glucagon from proglucagon. The aim of this study was to investigate the effects of chronic palmitate exposure on insulin pathways, GLP-1 secretion and glucagon synthesis in the GLUTag L-cell line. Cells were cultured in the presence/absence of palmitate (0.5 mM) for 24 h to mimic lipotoxicity. Palmitate treatment affected insulin-stimulated GLP-1 secretion, insulin receptor phosphorylation and IRS-1-AKT pathway signaling. In our model lipotoxicity induced extracellular signal-regulated kinase (ERK 44/42) activation both in insulin stimulated and basal conditions and also up-regulated paired box 6 (PAX6) and proglucagon expression (*Gcg*). Interestingly, palmitate treatment caused an increased glucagon secretion through the up-regulation of prohormone convertase 2. These results indicate that a state of insulin resistance could be responsible for secretory alterations in L-cells through the impairment of insulin-signaling pathways. Our data support the hypothesis that lipotoxicity might contribute to L-cell deregulation.

## 1. Introduction

Glucagon-like peptide-1 (GLP-1) is a peptide hormone, secreted by intestinal epithelial L-cells in response to hormonal, neural and nutrient stimuli. The most well known action of this hormone is to potentiate postprandial insulin release in a glucose-dependent manner [1,2,3]. GLP-1 regulates insulin and glucagon secretion from pancreatic islets and, with other gastrointestinal hormones such as GIP (glucose-dependent insulinotropic peptide), contributes to glucose homeostasis and metabolism regulation in healthy individuals [4,5].

In the light of these observations, in the past few years, it has been postulated that stimulation of endogenous GLP-1 secretion/production could constitute a powerful approach for the treatment of T2DM and obesity [6,7].

To date, it is known that the presence in the intestinal lumen of food or its macronutrients components (carbohydrates, fats and proteins), is the primary stimulus of GLP-1 secretion. Also other factors from the general blood circulation, such as hormones and cytokines, are able to both positively and negatively influence GLP-1 secretion [8,9]. Free fatty acids (FFAs) are able to potently stimulate GLP-1 secretion, as has been demonstrated both in vitro [10] and in vivo [11], more specifically, unsaturated fatty acids seem to be more potent stimulators of incretin secretion than saturated fatty acids [12,13]. At the level of intestinal L-cells, FFAs might interact with both the apical and basolateral (vascular) side. L-cells are polarized and differentially exposed to luminal and plasma constituents at their apical and basolateral membrane surfaces [14]; they are therefore able to respond to stimuli coming both from the gut lumen and the bloodstream [15,16,17]. Regarding hormonal stimuli, it has been reported that insulin stimulates GLP-1 secretion through the activation of the phosphatidylinositol 3 kinase (PI3K)-protein kinase B (AKT) and mitogen activated kinase (MAPK)-ERK1/2 pathways in murine and human L-cells [8]. Moreover, in these cells the insulin receptor (IR) exists in two isoforms, isoform A (IR-A) and isoform B (IR-B) [8], which could be activated by insulin, insulin-like growth factors or other mediators [18]; to date, the role of the IR and its isoforms on L-cell function is largely unknown.

Therefore, the secretory patterns of L-cells could consequently be influenced by both the alimentary stimuli coming from the digestive tract and the hormonal and metabolic perturbations that occur at the level of the circulatory system. In L-cells, chronic exposure to FFAs, such as palmitate, the most abundant fatty acid in the circulation of obese animals [19], induces apoptosis and impairs GLP-1 secretion with a significant alteration of the expression of enteroendocrine hormones and enteroendocrine–specific transcription factors [20,21,22]. Over the past few years, it has been demonstrated that chronic elevation of circulating FFAs, occurring in T2DM, could induce a state of insulin resistance in many tissues [23,24,25,26,27,28]. For example, it has been shown that lipotoxicity impairs insulin signaling and glucagon secretion in pancreatic alpha cells [29]; in addition it has been recently reported that insulin resistance, characterized by hyperinsulinemia, could affect proglucagon processing, inducing GLP-1 secretion in pancreatic alpha cells [30]. In L-cells, it has already been shown that a chronic treatment with high concentrations of insulin is able to induce an insulin resistance state that causes a reduction in basal and insulin-stimulated GLP-1 secretion [8]. Therefore, as already observed in other models, an insulin resistance state, induced by lipotoxicity, could impair insulin-mediated GLP-1 secretion in L-cells.

Interestingly, the existence of extrapancreatic glucagon produced by the gastrointestinal tract in totally pancreatectomized animals or humans has been reported [31,32,33,34,35,36]; intestinal L-cells, specifically, seem to be able to process proglucagon into glucagon through the action of prohormone convertase 2 (PC2), the proteolytic enzyme known to be predominantly expressed by pancreatic α-cells [37]. However, the molecular physiology of the intestinal glucagon production and possible effects of metabolic perturbations, such as lipotoxicity, should be investigated.

For all the above-mentioned reasons the aim of this study was, therefore, to investigate insulin signaling pathways and insulin stimulated GLP-1 secretion in L-cells chronically exposed to palmitate. Subsequently, in the light of recent data indicating the existence of intestinal glucagon production [31,32,38], we evaluated the expression of PC2 protein and then measured glucagon secretion. To test our hypothesis, we used GLUTag cells, a well-characterized murine model of GLP-1 secretion.

## 2. Results

### 2.1. Cell Viability and Lipid Accumulation of GLUTag Cells Treated with Palmitate

In order to evaluate palmitate cytotoxicity, we performed the MTT assay on GLUTag cells exposed to increasing palmitate concentrations (0.25, 0.50 and 1.00 mM) for different periods of time (12, 24 or 48 h).

**Figure 1:**
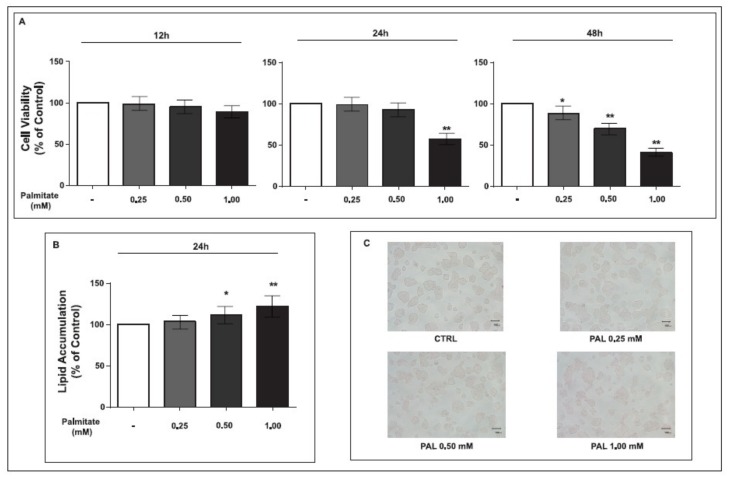
Effect of pre-exposure to palmitate on cell viability and lipid accumulation in GLUTag cells. **A**: MTT assay in GLUTag cells pre-exposed to palmitate (0.25, 0.50 and 1.00) after 12 h, 24 h and 48 h. Data are expressed as means ± standard error of 570 nM absorbance to % of control. * *p* < 0.05, ** *p* < 0.01, vs. control (*t*-test, *n* = 6). **B**: Nile Red staining in GLUTag cells pre-exposed to palmitate (0.25, 0.50 and 1.00 for 24 h). Data are expressed as means ± standard error of fluorescence to % of control. * *p* < 0.05, ** *p* < 0.01, vs. control (*t*-test, *n* = 6). **C**: Oil red O staining in GLUTag cells treated with palmitate (0.25, 0.50 and 1.00 for 24 h). A slight increase in Oil red O stained droplets (red) is visible in the cells treated with palmitate (0.50 and 1.00 mM) as compared with non-treated cells (40× magnification).

As shown in Figure 1, while 12 h of palmitate treatment did not affect cell viability at all of the tested concentrations, after 24 h of exposure, palmitate exerted a cytotoxic effect (cell viability = 57.17%, *p* < 0.01) only at the highest dose (1.00 mM). At 48 h, palmitate treatment was toxic at all the analyzed concentrations, in a dose-dependent manner (Figure 1A). Based on these results, we excluded the 48 h time point for further experiments concerning lipid accumulation.

After 12 h of treatment, we did not observe any statistically significant increase of lipid accumulation at any tested palmitate concentration, while lipid accumulation was evident in cells exposed to palmitate after 24 h of treatment at 0.50 mM and 1.00 mM, with a dose-dependent increase (Figure 1B). Oil Red O staining confirmed the dose-dependent increase of fat accumulation in the cytosol after 24 h of palmitate treatment (Figure 1C).

To perform the following experiments, we chose the dose-time combination of 0.5 mM for 24 h, in order to achieve a significant fat overload in the absence of any cytotoxic effect.

### 2.2. Chronic Palmitate Exposure Reduced Insulin-Induced GLP-1 Secretion

To determine the effect of a chronic exposure to palmitate on GLP-1 release, GLUTag cells were pre-treated with 0.5 mM palmitate or vehicle for 24 h. At the end of this period, cells were serum starved for 2 h, and subsequently incubated for 2 h in medium containing 25 mM glucose in the presence or absence of insulin (10^−9^ M).

**Figure 2:**
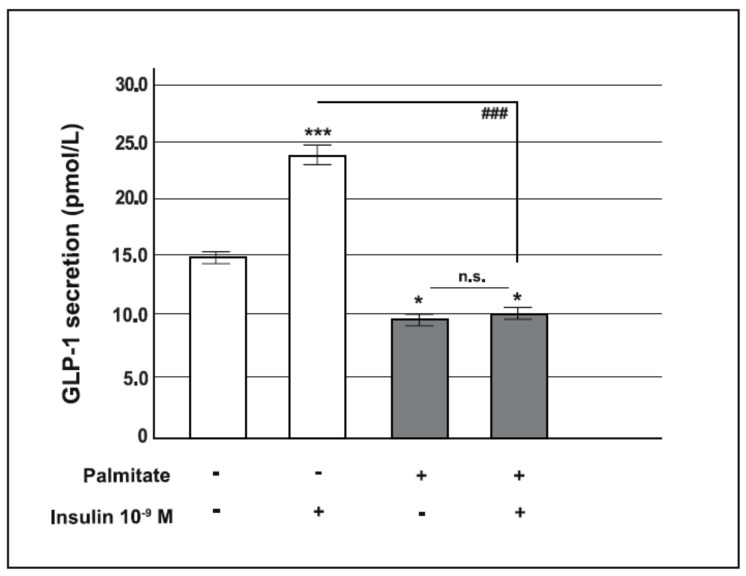
Effect of pre-exposure to palmitate on glucagon-like peptide-1 (GLP-1) secretion in GLUTag cells. Acute insulin-induced GLP-1 secretion in control cells (open bars) and in cells pre-exposed to 0.5 mM of palmitate for 24 h (gray bars). * *p* < 0.05, *** *p* < 0.001 vs. basal level in control group; ^###^ *p* < 0.001 vs. insulin stimulated control group, n.s. not significant (1-way ANOVA followed by Bonferroni test, *n* = 4); (+) means presence, (-) means absence.

As shown in Figure 2, in control cells, insulin significantly stimulated GLP-1 secretion (14.7 ± 0.4 vs. 23.4 ± 0.8; *p* < 0.001). Conversely, in cells chronically exposed to palmitate a small but significant decrease in GLP-1 release was observed in the absence of insulin compared to control cells (14.7 ± 0.4 vs. 9.6 ± 0.3; *p* < 0.05); moreover, in these cells GLP-1 secretion did not increase after insulin stimulation, thus the insulin stimulatory effect on GLP-1 secretion was completely abrogated by palmitate treatment (23.4 ± 0.8 vs. 10.1 ± 0.4; *p* < 0.001).

### 2.3. Palmitate Impaired IR Phosphorylation and the IRS-1/AKT Pathway

**Figure 3:**
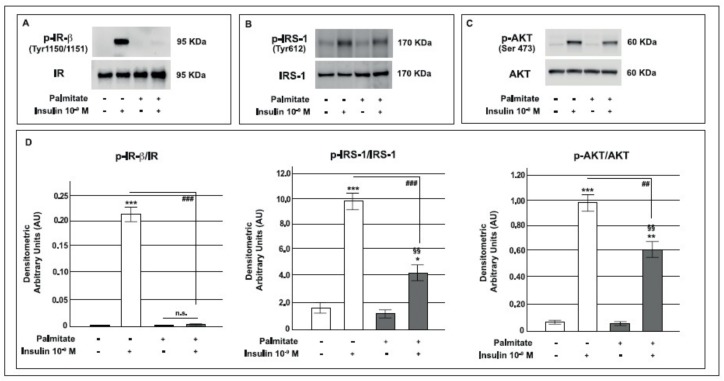
Effect of pre-exposure to palmitate on IR phosphorylation and the IRS-1/AKT pathway in GLUTag cells. Representative immunoblot from control and palmitate GLUTag treated cells (0.5 mM for 24 h) acutely stimulated with insulin 10^−9^ M for 5 min for: **A**: immunoprecipitation of the total IR (Tyr1150/1151 on the β subunit) (p-IR-β) and total IR; **B**: p-IRS-1 (Tyr612) and total IRS-1 (IRS-1); **C**: p-AKT (Ser 473) and total AKT (AKT); **D**: corresponding densitometric analysis in control cells (open bars) and in cells exposed to palmitate (gray bars). * *p* < 0.05, ** *p* < 0.01, *** *p* < 0.001 vs. basal control; ^##^ *p* < 0.01, ^###^ *p* < 0.001 vs. insulin stimulated control group; ^§§^ *p* < 0.01 vs. palmitate; n.s. not significant (1-way ANOVA followed by Bonferroni test, *n* = 3); (+) means presence, (-) means absence.

In order to investigate the molecular mechanisms by which palmitate altered insulin-stimulated GLP-1 secretion from GLUTag cells, we analyzed some mediators of the intracellular insulin pathway. We first examined the activation of the IR and insulin metabolic pathway. As shown in Figure 3, in control cells acute stimulation with 10^−9^ M insulin for 5 min induced a significant increase in the tyrosine phosphorylation of the IR β subunit, whereas in palmitate pre-exposed cells, the insulin effect on IR phosphorylation was completely abrogated (Figure 3A).

Subsequently, we studied IRS-1 phosphorylation (Tyr612) [p-IRS-1] by using Western blot analysis. As presented in Figure 3, in control cells, insulin significantly stimulated IRS-1 phosphorylation; in contrast, in palmitate pre-exposed cells this effect was markedly reduced (Figure 3B). Since p-IRS-1 activates PI3K by binding to its 85-kDa subunit, we performed a co-immunoprecipitation for IRS-1-PI3K p-p85α (Tyr508) and found a significant reduction of insulin activation in palmitate pre-treated cells (Figure S1). Finally, we evaluated AKT phosphorylation (Ser473) [p-AKT] and, as shown in Figure 3, in palmitate pre-exposed cells insulin-induced AKT phosphorylation was significantly decreased compared to control cells (Figure 3C).

These results suggest that in GLUTag cells chronic exposure to palmitate induced a state of insulin resistance by blocking the IRS-1/AKT pathway.

### 2.4. Palmitate Increased the MAPK Pathway

**Figure 4:**
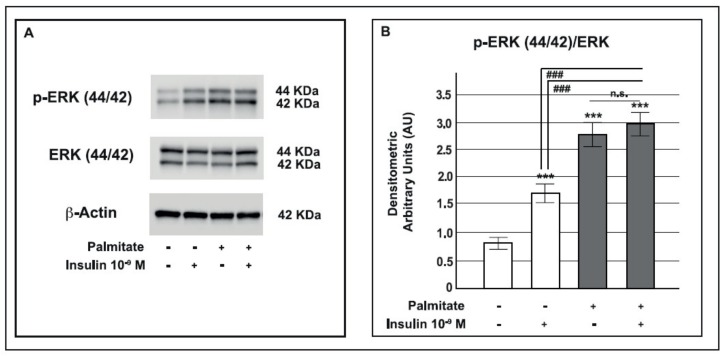
Effect of pre-exposure to palmitate on MAPK pathway activation in GLUTag cells. **A**: representative Western blot for p-ERK 44/42, total ERK 44/42 (ERK) and β-actin from control and palmitate GLUTag treated cells (0.5 mM for 24 h) acutely stimulated with insulin 10^−9^ M for 5 min. **B**: corresponding densitometric analysis in control cells (open bars) and in cells exposed to palmitate (gray bars). *** *p* < 0.001 vs. basal control; ^###^ *p* < 0.001 vs. insulin stimulated control group; n.s. not significant (1-way ANOVA followed by Bonferroni test, *n* = 3); (+) means presence, (-) means absence.

To investigate the effect of palmitate treatment on the MAPK pathway, we evaluated ERK 44/42 insulin-stimulated phosphorylation (p-ERK 44/42). As shown in Figure 4, in control GLUTag cells, the ERK 44/42 phosphorylated form significantly increased in response to a 5 min acute 10^−9^ M insulin stimulation. In cells cultured with palmitate, the basal ERK 44/42 phosphorylated form was markedly higher than in control cells and insulin maintained this activation.

### 2.5. Palmitate Up-regulated the IR-A/IR-B Ratio

**Figure 5:**
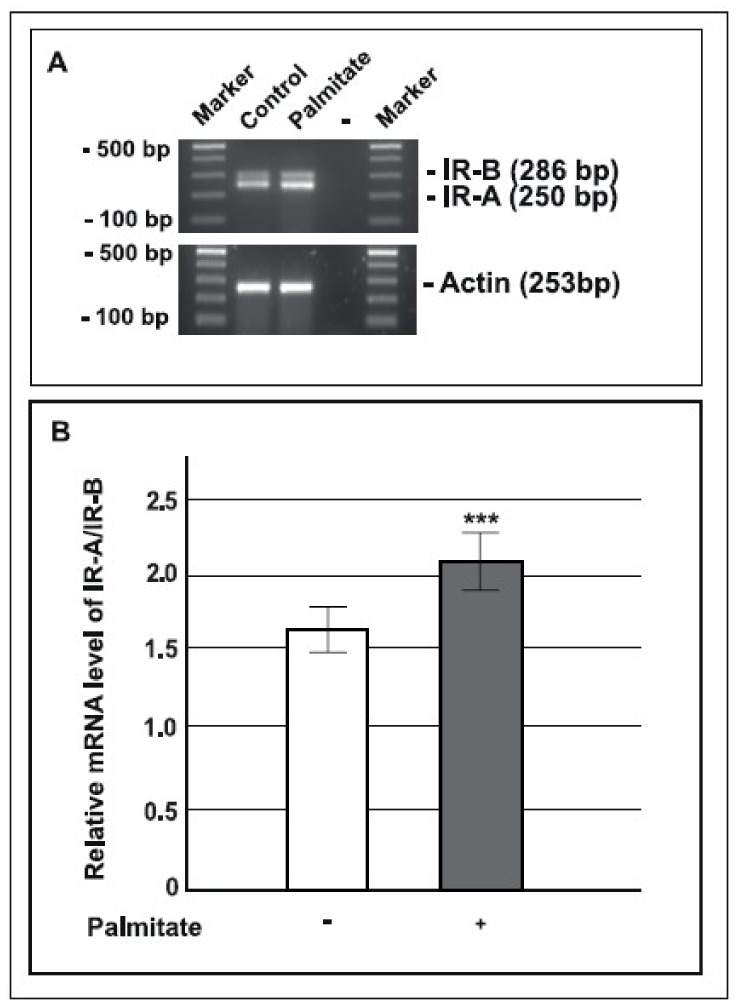
IR-A and IR-B mRNA expression in GLUTag cells. **A**: representative agarose gel image for RT-PCR analysis of IR-B (286 bp) and IR-A (250 bp) mRNA expression in control and palmitate treated cells (0.5 mM for 24 h). “-“ represents PCR reaction without added template. **B**: statistical data showing the mRNA ratio of IR-A/IR-B in control cells (open bars) and in cells exposed to palmitate (gray bars) (*n* = 3, *** *p* < 0.001 compared to control); (+) means presence, (-) means absence.

The IR gene undergoes differential splicing that generates two IR isoforms, IR-A and IR-B. Since it has been reported that the IR-A isoform could up-regulate p-ERK 44/42 protein expression [39], to better investigate basal ERK 44/42 phosphorylation induced by chronic palmitate exposure we analyzed the mRNA expression levels of IR-A and IR-B by RT-PCR and, as shown in Figure 5, we found that the IR-A to IR-B mRNA ratio was significantly higher in cells cultured with palmitate compared with control cells.

In order to exclude the involvement of G Protein-Coupled Receptors (GPCRs) on the MAPK pathway activation, we analysed GPR119 and GPR120 protein expression by Western blot and we did not observe any significant difference in palmitate treated cells with respect to control (Figure S2).

These data suggest that the activation of the MAPK pathway, observed in palmitate treated cells, was probably due to the increase of IR-A isoform expression.

### 2.6. Palmitate Increased PAX6 and Proglucagon Expression

**Figure 6:**
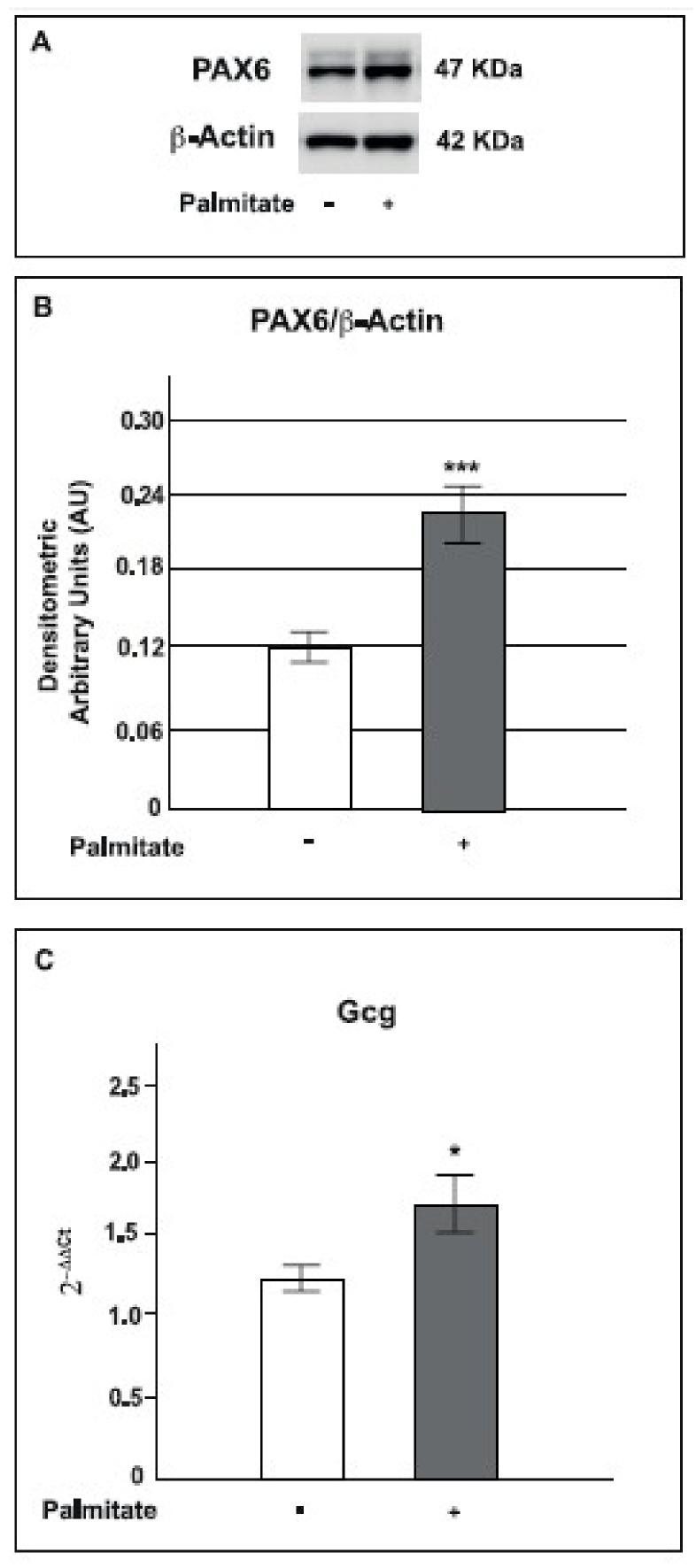
Effect of palmitate on PAX6 and proglucagon expression in GLUTag cells. **A**: representative Western blot for PAX6 and β-actin from control and palmitate GLUTag treated cells (0.5 mM for 24 h). **B**: corresponding densitometric analysis in control cells (open bars) and in cells exposed to palmitate (gray bars). *** *p* < 0.001 vs. control (1-way ANOVA followed by Bonferroni test, *n* = 3). **C**: box plot of proglucagon (*GcG*) mRNA expression in control and palmitate treated cells (0.5 mM for 24 h). Values on the y-axis are reported as 2^−ΔΔ*C*t^. Statistical significance was evaluated by *t*-test (*n* = 3, * *p* < 0.05 compared to control); (+) means presence, (-) means absence.

Considering that the MAPK signaling pathway could regulate PAX6, a transcription factor required for proglucagon (*Gcg*) gene expression in the pancreas and intestine [40,41,42], PAX6 protein expression was also investigated. As shown in Figure 6, in GLUTag cells, after 24 h of palmitate exposure, PAX6 protein expression was significantly increased compared to control cells (Figure 6A,B).

Because PAX6 regulates proglucagon (*Gcg*) gene expression, we also measured *Gcg* gene expression by Real-time PCR in GLUTag cells treated with palmitate. As expected, *Gcg* gene expression was significantly increased in cells cultured for 24 h with palmitate (0.5 mM) compared to control cells (Figure 6C).

These results indicate that chronic palmitate exposure activates the MAPK pathway, which in turn induces transcription factor PAX6 increased protein expression, and subsequent proglucagon gene up-regulation.

### 2.7. Palmitate Increased Prohormone Convertase PC2 Expression and Glucagon Secretion

The proglucagon precursor gives rise to numerous peptides belonging to the glucagon superfamily of hormones [16]. Tissue-specific post-translational processing by prohormone convertase 1/3 (PC1/3) or PC2 results in a different profile of proglucagon-derived peptides (PGDPs) in these tissues. Thus, as a result of cleavage by PC2, the major bioactive PGDP being produced in pancreatic α-cells is glucagon while post-translation processing by PC1/3 in the intestine preferentially leads to GLP-1 and GLP-2 [37,43,44]. In recent years the existence of extrapancreatic glucagon secreted by the gut in humans has been demonstrated [32,45,46]. PC2 is also expressed by enteroendocrine cells and these cells seem to be able to process proglucagon to glucagon by using PC2 and to secrete glucagon under specific conditions [20,31,32,47,48,49].

Based on these findings, to better investigate *Gcg* expression increase, we studied PC2 expression and glucagon secretion in our system.

**Figure 7:**
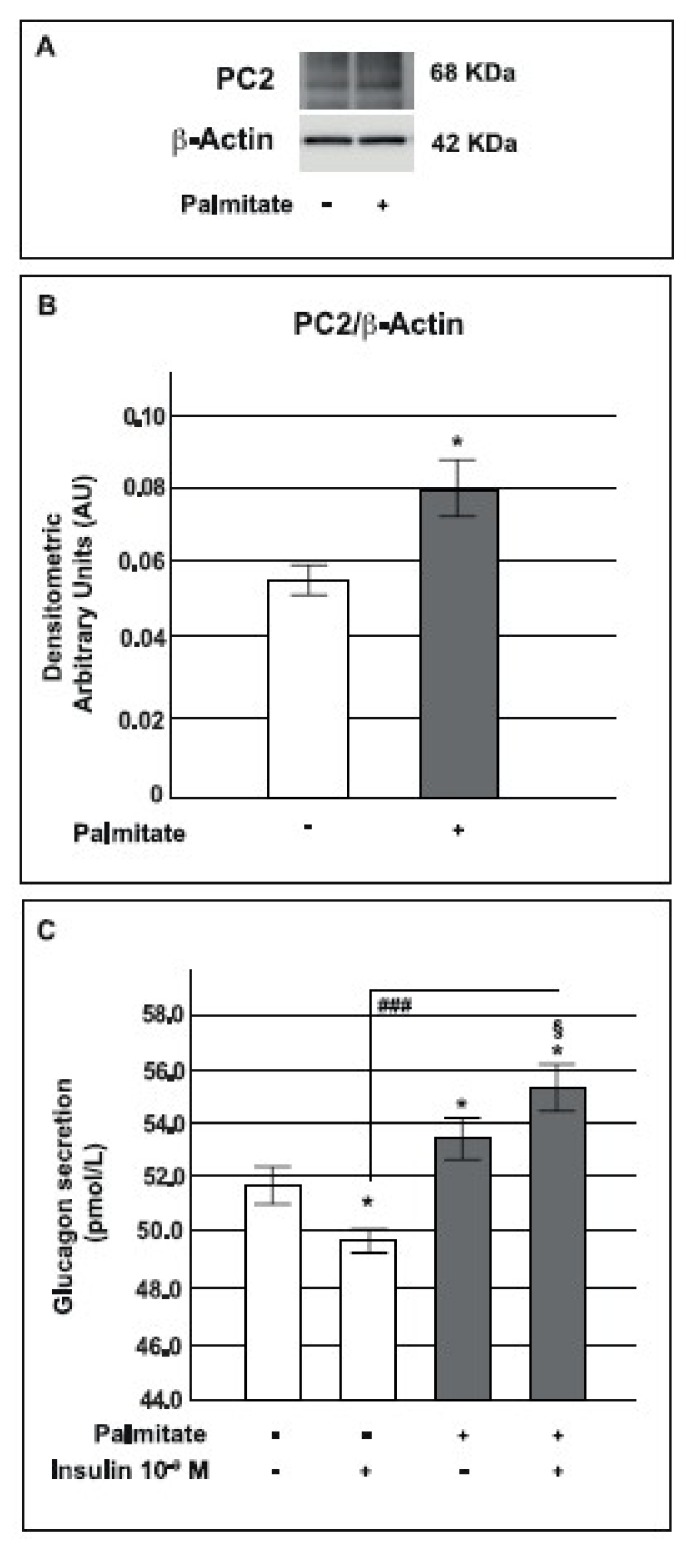
Effects of pre-exposure to palmitate on PC2 protein expression and glucagon secretion. **A**: representative Western blot for PC2 and β-actin from control and palmitate GLUTag treated cells (0.5 mM for 24 h). **B**: corresponding densitometric analysis in control cells (open bars) and in cells exposed to palmitate (gray bars). * *p* < 0.05 vs. control (1-way ANOVA followed by Bonferroni test, *n* = 3). **C**: glucagon secretion in control cells (open bars) and in cells pre-exposed to 0.5 mM of palmitate for 24 h (gray bars). * *p* < 0.05 vs. basal level in the control group; ^###^ *p* < 0.001 vs. insulin stimulated control group; § *p* < 0.05 vs basal level in the palmitate group (1-way ANOVA followed by Bonferroni test, *n* = 4); (+) means presence, (-) means absence.

We measured the protein expression of PC2 in GLUTag cells by Western blot analysis and, as presented in Figure 7, we observed that under our experimental conditions, the expression of the active form of PC2 was significantly enhanced in cells exposed to palmitate compared to control cells (Figure 7A,B).

In the light of these results, we decided to investigate glucagon secretion in our model. As shown in Figure 7, in cells chronically treated with palmitate, basal glucagon secretion was slightly increased with respect to control cells and, moreover, the inhibitory effect of insulin on glucagon secretion was almost abrogated (Figure 7C).

These data indicated that chronic exposure to palmitate by up-regulating PC2 expression stimulated the synthesis of glucagon by GLUTag cells.

### 2.8. Palmitate Up-Regulated the MAPK Pathway and Its Downstream Targets

**Figure 8:**
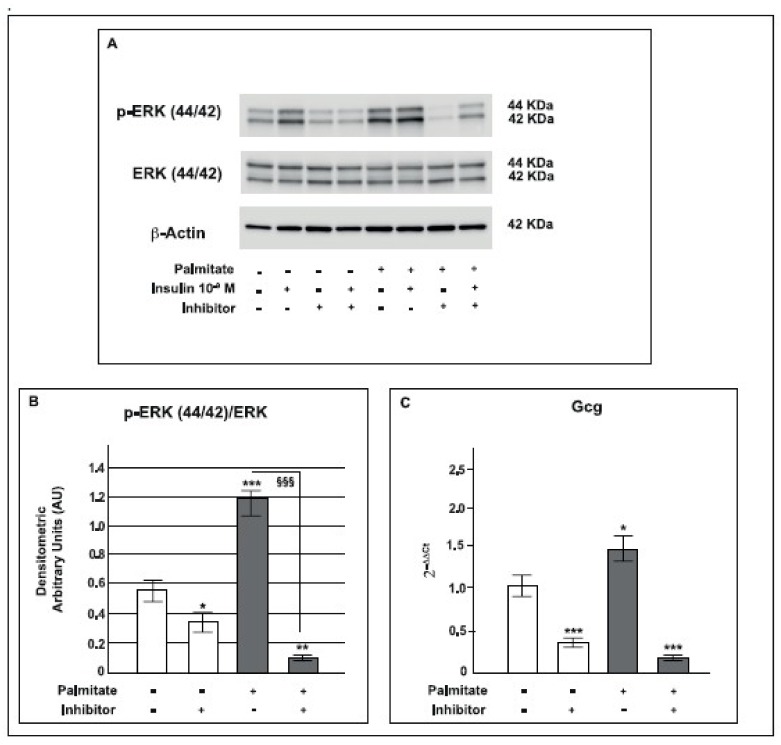
Effect of MAPK pathway inhibition on proglucagon gene expression in GLUTag cells pre-exposed to palmitate. **A**: representative Western blot for p-ERK 44/42, ERK 44/42 and β-actin from control and palmitate GLUTag treated cells (0.5 mM for 24 h) in the absence or presence of the U0126 (MAPK Inhibitor). **B**: corresponding densitometric analysis in control cells (open bars) and in cells exposed to palmitate (gray bars). * *p* < 0.05, ** *p* < 0.01, *** *p* < 0.001 vs. control; ^§§§^ *p* < 0.001 vs. palmitate (1-way ANOVA followed by Bonferroni test, *n* = 3). **C**: Box plot of proglucagon (*GcG*) mRNA expression in control cells (open bars) and in 0.5 mM palmitate treated cells for 24 h (gray bars) in the absence or presence of U0126. Values on the y-axis are reported as 2^−ΔΔ*C*t^. Statistical significance was evaluated by *t*-test (*n* = 3, * *p* < 0.05, *** *p* < 0.001 vs. control); (+) means presence, (-) means absence.

**Figure 9:**
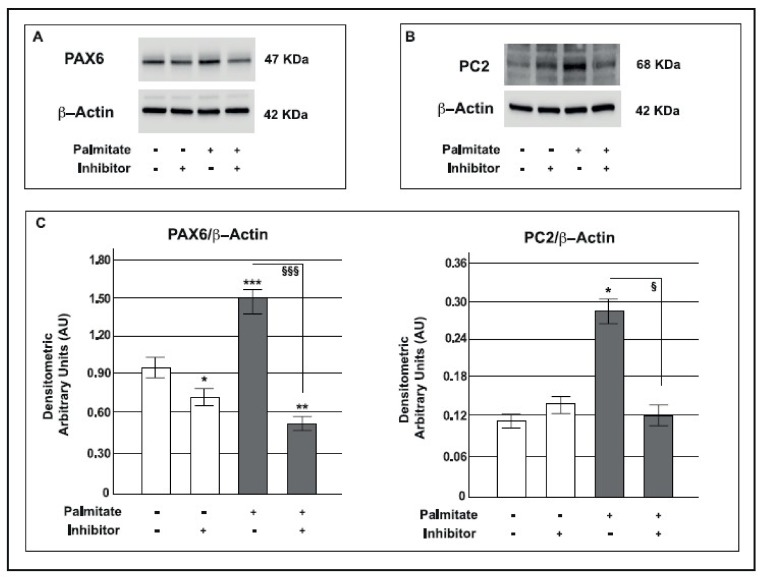
Effects of MAPK pathway inhibition on PAX6 and PC2 protein expression in GLUTag cells pre-exposed to palmitate. Representative Western blot from control and palmitate GLUTag treated cells (0.5 mM for 24 h) in the absence or presence of U0126 (MAPK Inhibitor) for: **A**: PAX6 and β-actin; **B**: PC2 and β-actin; **C**: corresponding densitometric analysis in control cells (open bars) and in cells exposed to palmitate (gray bars). * *p* < 0.05, ** *p* < 0.01, *** *p* < 0.001 vs. control; *§ p* < 0.05, ^§§§^ *p* < 0.001 vs. palmitate (1-way ANOVA followed by Bonferroni test, *n* = 3); (+) means presence, (-) means absence.

To confirm the role of MAPK in modulating PAX6 and *Gcg* expression, a specific block of the MAPK pathway was performed using the inhibitor U0126, which selectively inhibits MEK1 activity, the ERK 44/42 controller in the MAPK pathway. As expected, U0126 inhibited both ERK 44/42 (Figure 8A,B) and PAX6 protein expression (Figure 9A). Moreover, in GLUTag cells U0126 was able to significantly reduce *Gcg* gene expression (Figure 8C). Interestingly, PAX6 was previously found to be able to regulate the expression of PC2 and its molecular chaperone, 7B2 [50]. Consequently, in order to verify if ERK 44/42 activity and its downstream target PAX6 were able to affect PC2 expression we investigated this prohormone in our model and found that the presence of the MAPK inhibitor significantly reduced PC2 protein expression in palmitate treated cells (Figure 9B).

These data indicate the clear relationship between MAPK pathway, PAX6, PC2 and *Gcg* expression in our model*;* MAPK inhibition induced by U0126 was able to reverse the effect generated by chronic palmitate treatment.

## 3. Discussion

In this work we studied the effect of a chronic exposure to high palmitate levels on insulin-induced GLP-1 secretion, insulin signal pathways and glucagon release in intestinal L-cells. Specifically, we focused on the stimuli coming from the circulatory system, focusing our attention on the metabolic perturbations that predominantly act at the level of the basolateral compartment of L-cells. We used the GLUTag cell line, a stable, immortalized relatively differentiated murine enteroendocrine cell line that expresses the proglucagon gene and that secretes glucagon-like peptides in a regulated manner [51,52]. GLUTag cells are one of the best models for studying L-cells because native L-cells are very scarce and dispersed as individual cells along the gastrointestinal tract and cannot be isolated to provide homogenous L-cell culture [53], moreover, they recapitulate the response of primary L-cells to physiological and pharmacological GLP-1 secretagogues [52,54].

Our data provide evidence that chronic treatment with palmitate significantly inhibited insulin-stimulated GLP-1 release from GLUTag cells and that this secretory alteration is probably associated with an impairment of the IRS-1/AKT signaling pathway. In contrast, the p-ERK 44/42, PAX6 and proglucagon pathway was up-regulated already in the “basal” level (i.e., in the absence of insulin stimulation) in cells exposed to palmitate. This activation seemed to be correlated with an increased IR-A/IR-B ratio and, most importantly, was associated with increased PC2 expression and glucagon secretion in GLUTag cells exposed to palmitate.

It is known that patients with T2DM frequently exhibit elevated levels of plasma FFAs [52,54] associated with insulin resistance of many cell types and ectopic lipid deposition [55]. Since palmitate is the most abundant saturated FFA [56,57,58] that mainly increases during diabetes, both in humans and in experimental animal models [59], in this study we used it to mimic diabetic hyperlipidemia and insulin resistance in an in vitro model of intestinal L-cells. Our data are in agreement with previous reports that explored intestinal lipid accumulation in several metabolic disorders such as obesity and diabetes [60,61,62].

It was previously reported that lipotoxicity, induced by high levels of palmitate, was able to reduce GLP-1 secretion and caused endoplasmic reticulum stress and apoptosis in GLUTag cells [10,63]. In our model, we observed that treatment with high palmitate concentrations (0.5 mM for 24 h) inhibited insulin–stimulated GLP-1 secretion and induced a state of insulin resistance at the level of the IRS-1/AKT pathway.

Although the role of insulin on GLP-1 release has not yet been fully clarified, insulin receptors have been described in intestinal L-cells [8,64,65] and their loss has been shown to be associated with impaired GLP-1 secretion and altered expression of intestinal epithelial genes related to glucose uptake and metabolism [8,53,65]. The activated form of the IR is able to phosphorylate both IRS-1-PI3K-AKT and MAPK pathways in L-cells. Lim et al. [8] demonstrated the direct effect of chronic insulin treatment on GLP-1 secretion showing that an induced state of insulin resistance was able to markedly reduce the activation of both AKT and ERK1/2 [8]. Moreover, it has been reported that phosphorylation of the IR and AKT significantly decreased after incubation with palmitate also in NCI-H716 human L-cells, and metformin treatment reversed these effects and then enhanced GLP-1 secretion [64]. In agreement with these data, in our study, lipotoxicity, by inducing insulin resistance at the level of the IR/IRS-1/AKT pathway, reduced acute insulin stimulated GLP-1 secretion. Moreover, our results demonstrate that lipotoxicity-mediated insulin resistance could impair GLP-1 secretion acting at the level of proglucagon processing. Under our experimental conditions, increased proglucagon gene expression, mediated by MAPK/PAX6 activation, was associated with an increase of glucagon secretion rather than GLP-1 secretion and this effect could be determined by PC2 up-regulation.

A very interesting and original finding of this study was that in GLUTag cells cultured with palmitate, the MAPK intracellular pathway was activated also in the absence of insulin stimulation.

The ERK pathway is a central MAPK pathway that is triggered by various stimuli [66,67] and coordinates a wide range of cell functions, including cell proliferation, differentiation, gene expression and apoptosis [68,69]. In our study, we focused our attention on ERK 44/42 activation, a key protein kinase in the ERK1/2 signaling pathway and we found that its active form (phosphorylated) was also increased at the basal state in GLUTag cells cultured with palmitate.

Previous studies that already addressed this issue, found that insulin resistance impaired GLP-1 secretion form GLUTag cells by down-regulating the MAPK intracellular pathway [8]. It is likely that the discordance between our data and these observations can be simply explained by the substantial differences in the models used; indeed, in the study of Lim and colleagues, the authors used supra-physiological concentrations of insulin (10^−7^ M for 24 h) to induce a state of insulin-resistance. In our study, we chose to use high palmitate concentrations, which in our opinion is a more physiological inducer of insulin resistance. Moreover, consistent with the results we obtained, a large body of evidence has already suggested that elevated FFAs and their lipid intermediates are able to cause an abnormal activation of the MAPK pathway, which might result in insulin resistance [70,71,72].

In recent years it has been demonstrated that up-regulation of IR-A, characterized by a high IR-A/IR-B ratio, was able to activate the ERK 44/42 pathway in diabetic mice [39]. In agreement with these data, in our model, we observed that the IR-A/IR-B ratio was increased in GLUTag cells after palmitate treatment with respect to control cells. This result indicates that palmitate treatment might play a role in the overexpression of IR-A and in the consequent activation of the ERK pathway in GLUTag cells.

It has been previously reported that the MAPK pathway mediates many intracellular processes, including activation of some transcription factors; among these, PAX6 has important functions in the development of several tissues, such as the central nervous system, eye, nose, and pancreas [73,74,75,76]. In order to analyze the effects of the ERK pathway activation in our model, we studied PAX6 protein expression and found that it was significantly higher in cells treated with palmitate. Drucker group [42] showed that PAX6 is expressed in enteroendocrine cells and regulates proglucagon gene expression [40]. Based on these data we analyzed proglucagon gene expression and found that it was significantly increased in GLUTag cells cultured with palmitate, as previously reported [77].

Although it is known that in physiological conditions PC1/3 splices proglucagon to GLP-1 in L-cells [78,79], while PC2 processes it to glucagon in pancreatic α-cells [37,80], in the last few years many studies have shown that pancreatic α-cells are able to express PC1/3 and to produce GLP-1 under particular conditions such as metabolic stress, inflammation and insulin resistance state [30,81]. Interestingly, the existence of immunoreactive extrapancreatic glucagon produced by intestinal cells in totally pancreatectomized animals and humans has been amply demonstrated [31,32,33,34,35,36]; in particular Lund et al. demonstrated an increase in circulating glucagon levels following oral ingestion of glucose in totally pancreatectomized patients with type 2 diabetes [32]. Based on these studies, we hypothesized that metabolic alterations, such as chronic palmitate exposure, could impair proglucagon processing with a subsequent increase of PC2 expression and glucagon secretion in L-cells. Indeed, recent studies have demonstrated that PC2 was expressed in enteroendocrine cells of the human gut [31,46] and that its expression was increased in type 2 diabetic individuals [48]. In agreement with these observations, in our model both the protein expression of PC2 and glucagon were highly increased in GLUTag cells treated with palmitate and these results clarified the augmented proglucagon gene expression.

To further confirm the relationship between the up-regulated MEK/ERK, PAX6 and proglucagon pathway, we tested U0126, a specific inhibitor of MEK, the ERK 44/42 controller in the MEK/ERK pathway [82,83]. Following U0126 treatment, as expected the up-regulated expression of p-ERK 44/42 was significantly decreased in GLUTag cells pre-exposed to palmitate. Interestingly, U0126 was also able to reverse the increased protein expression of PAX6 and proglucagon gene expression; as a result of this effect also PC2 protein expression was reduced under the same experimental conditions. These results confirm that the increased expression of PAX6 and proglucagon in GLUTag cells treated with palmitate was induced by the MEK/ERK pathway, as well as PC2 expression.

In summary, our data indicate that GLUTag cells, an in vitro model of L-cells, cultured for 24 h in the presence of high palmitate concentrations (0.5 mM) developed insulin resistance as a result of intracellular lipid accumulation, confirming the already evidenced harmful association between lipotoxicity and impaired insulin sensitivity. Specifically, these cells showed an impairment of the IRS-1/AKT insulin signaling pathway, which likely controls insulin stimulated GLP-1 secretion and an activation of the MAPKs pathway already in the basal state. This is likely through an increased expression of the IR-A/IR-B ratio, leading to increased PAX6, proglucagon and especially PC2 expression. The observed final effect was the rise of glucagon secretion in a cellular model originally developed to secrete GLP-1.

In conclusion, these findings have shed new light on the roles and the functions of the IR in GLP-1 secretion and on the molecular mechanisms associated with the dysfunction of L-cells after chronic exposure to palmitate. These data, although produced in an in vitro model, give rise to speculations based on observations obtained in diabetic patients; our data, may, therefore, contribute to explain the abnormalities in L-cell function and the hyperglucagonemia that typically occur in diabetes. The contribution of intestinal glucagon in diabetic hyperglucagonaemia needs to be further studied but we think that, although pancreatic alpha cells definitely play the major role in the development of hyperglucagonemia in diabetes, our findings clearly show that also increased glucagon secretion produced by intestinal L-cells contributes to worsening of this state. Future studies using in vivo models will be able to support our findings.

## 4. Materials and Methods

### 4.1. Cell Culture

Murine GLUTag cells (courtesy of F.M. Gribble University of Cambridge, with permission from D.J. Drucker University of Toronto) [51,54,84] were grown in DMEM (Dulbecco’s modified Eagle’s medium) containing 5.6 mmol/L glucose (Sigma-Aldrich, Saint Louis, MO, USA), supplemented with 10% heat-inactivated dialyzed fetal bovine serum (Gibco, Thermo Fisher Scientific, Rodano, MI, Italy), penicillin, streptomycin and L-glutamine (Sigma-Aldrich, Saint Louis, MO, USA). The cells were passaged once a week and were maintained at 37 °C in a humidified incubator gassed with 5% CO_2_. Most of the experiments were performed with cells at passage 12–22, however, the response of cells was similar for passages 10–25.

### 4.2. Chronic Exposure to Palmitate

Palmitate solution (Sigma-Aldrich, Saint Louis, MO, USA) was prepared as previously reported [85] and was diluted in culture medium.

To identify the best biological response to the treatment, cells were exposed to increasing concentrations of palmitate (0.25, 0.50 and 1.00 mM) for different periods of time (12, 24 and 48 h).

### 4.3. MTT Cell Viability Assay

Cytotoxicity of palmitate to GLUTag cells was assessed by the MTT assay (Sigma-Aldrich, Saint Louis, MO, USA) as previously reported [85]. Optical absorbance was determined at 570 nm with a microplate spectrophotometer (Victor X3 Multilabel Plate Reader, PerkinElmer, Waltham, MA, USA).

### 4.4. Evaluation of Lipid Accumulation by Nile Red and Oil Red O Staining

Total intracellular lipid content was evaluated by Nile Red staining [86] (Sigma-Aldrich, Saint Louis, MO, USA), as previously reported [85]. Fluorescence was determined at an excitation of 488 nm and emission of 535 nm with a microfluorimeter (Victor X3 Multilabel Plate Reader PerkinElmer, Waltham, MA, USA). All the obtained values were normalized on cell proliferation by the Crystal Violet assay.

Fat staining with Oil Red O was performed as previously described [87]. GLUTag cells were fixed with 10% formaldehyde for 2 h and then stained with Oil Red O for 20 min, followed by washing with distilled water.

### 4.5. Insulin Stimulation

GLUTag cells were implanted and after 24 h from seeding were incubated for 24 h in complete DMEM in the presence or absence of 0.5 mM palmitate. After palmitate exposure, cells were serum starved with medium containing 1% BSA for 2 h before insulin stimulation (Sigma-Aldrich, Saint Louis, MO, USA). To test the effects of insulin stimulation, we conducted preliminary experiments with increasing doses of insulin (10^−10^ to 10^−8^ M) for 5 min in control cells and evaluated IRS-1 phosphorylation (Tyr612), by Western blot (Santa Cruz Biotechnology, Dallas, TX, USA). We chose the dose of 10^−9^ M insulin because this condition induced statistically significant activation of IRS-1; subsequent experiments were therefore performed under these conditions.

To test the insulin stimulation persistence, we evaluated AKT phosphorylation (Ser473) after 2 h of 10^−9^ M insulin exposure and we found that the insulin-mediated AKT activation was lost (Figure S3).

In a specific set of experiments to thoroughly evaluate the role of MAPK in PAX6 and proglucagon expression, a specific MEK inhibitor U0126 (Promega, Milan, Italy) was added at a concentration of 25 μmol/L [29] for 30 min prior to insulin stimulation or 24 h [88,89,90].

### 4.6. GLP-1 and Glucagon Secretion

To study GLP-1 and glucagon secretion, the cells were grown in the presence or absence of 0.5 mM palmitate for 24 h. At the end of treatment, cells were serum starved for 2 h and then were washed with Phosphate Buffered Saline (PBS) (Sigma-Aldrich, Saint Louis, MO, USA). Experiments were performed, as previously reported [8], by incubating the cells in a medium containing 25 mM glucose and 1% BSA in the presence or absence of insulin (10^−9^ M) for 2 h. At the end of the incubation period, media were collected in tubes containing aprotinin (0.1 mg/L) (Sigma-Aldrich, Saint Louis, MO, USA) and kept frozen at −20 °C for subsequent analysis. The cells were lysed in Radio-Immunoprecipitation Assay (RIPA) buffer and the lysates were analyzed for total protein content to control for the number of cells. The GLP-1 levels in the supernatant were measured using an ELISA kit for the quantitative determination of bioactive GLP-1 (7–36) (GLP-1 Active ELISA kit; ALPCO Diagnostic) as previously described [15]. The glucagon levels were measured using a specific ELISA kit (Mercodia Glucagon; Uppsala, Sweden) according to the manufacturer’s instructions.

### 4.7. Cell Lysis, Immunoprecipitation and Western Blot Analysis

At the end of the culture period, the cells were lysed in ice-cold modified RIPA buffer.

In some experiments, immunoprecipitation or co-immunoprecipitation was performed; in others, crude lysates were used.

Proteins A or G-Sepharose (GE Healthcare Life Sciences, Uppsala, Sweden) were incubated with 2–4 μg of the specific antibody, the IR β-subunit (IR-β) for immunoprecipitation and total IRS-1 for co-immunoprecipitation (Santa Cruz Biotechnology, Dallas, TX, USA), at 4 °C under constant rotation for 2 h and then overnight with cell lysates. Immunoprecipitates were subjected to SDS-PAGE and then analyzed by immunoblotting. Specifically, membranes with IR-β resolved proteins were immunoblotted with an anti phospho-insulin receptor β (Tyr1150/1151) (Cell Signaling Technology, Danvers, MA, USA) while membranes with IRS-1 resolved protein were immunoblotted with an anti PI3K phospho-p85α (Tyr508) (Santa Cruz Biotechnology, Dallas, TX, USA) to study the specific phosphorylations.

For Western blot analysis, cell lysates were analyzed as previously described [91]. Proteins of interest were detected with the following specific antibodies: anti-phospho-IRS-1 (Tyr612), anti total IRS-1, anti GPR119, GPR120 and anti PC2 (Santa Cruz Biotechnology, Dallas, TX, USA); anti-phospho-AKT (Ser473), anti-total AKT, anti-phospho-ERK 44/42 (Thr202/Tyr204) anti-ERK 44/42 (Cell Signaling Technology, Danvers, MA, USA); anti PAX6 (R&D System, Abingdon, UK) and anti β-Actin (Sigma-Aldrich Saint Louis, MO, USA).

Immunoblot signals were visualized using an Odissey Fc System infra-red scanner (LI-COR Biosciences, Lincoln, NE, USA). The densitometric analyses were performed using Odissey software Image Studio Lite Ver 5.2 (LI-COR Biosciences, Lincoln, NE, USA).

### 4.8. Total RNA Isolation, Reverse Transcription and Real-time PCR

Total RNA was extracted by using TRIzol reagent (Thermo Fisher Scientific, Rodano, MI, Italy) according to the manufacturer’s instructions and quantified by spectrophotometry.

In order to analyze insulin receptor isoforms (*Insr*), 1 μg of total RNA was converted to complementary DNA (cDNA) by reverse transcription using SuperScript III and Oligo dT reagents (Thermo Fisher Scientific, Rodano, MI, Italy). Two isoforms of *Insr* transcripts (NM_001330056) were obtained by reverse transcription polymerase chain reaction (RT-PCR) with primers for the flanking exons 10 and 12 of *Insr* using Platinum Green Hot Start PCR (Thermo Fisher Scientific, Rodano, MI, Italy) according to the manufacturer’s instructions, actin beta (*Actb Actβ*, NM_007393) was used as endogenous control. Aliquots of each amplified were analyzed by electrophoresis on 2/2.5% agarose gels and visualized by SYBR Safe DNA gel stain (Thermo Fisher Scientific, Rodano, MI, Italy).

Quantitative Real-time PCR was performed using Power SYBR^®^ Green RNA-to-CT™ 1-Step Kit (Thermo Fisher Scientific, Rodano, MI, Italy), briefly, 50 ng of total RNA was retrotranscribed and amplified in a single step according to the manufacturer’s protocol. Proglucagon (*Gcg*, NM_008100) gene expression was evaluated and glyceraldehyde-3-phosphate dehydrogenase (*Gapdh*, NM_001289726) was used as reference gene. Each experiment was performed in biological triplicate and gene expression changes were analyzed with the 2^−ΔΔ*C*t^ method.

### 4.9. Statistical Analysis

All data are presented as mean ± SEM. Data were analyzed by Student’s *t-*test or ANOVA, followed by appropriate *post hoc* testing. A *p* value less than 0.05 was considered statistically significant.

## Supplementary Materials

Supplementary materials can be found at http://www.mdpi.com/1422-0067/19/12/3791/s1. Figure S1: Effect of pre-exposure to palmitate on PI3K activation in GLUTag cells; Figure S2: Effect of pre-exposure to palmitate on GPR119 and GPR120 protein expression in GLUTag cells; Figure S3: Time course of p-AKT insulin-stimulation in GLUTag cells.
