# Supplementary material for: Chronic Exposure to Palmitate Impairs Insulin Signaling in an Intestinal L-cell Line: A Possible Shift from GLP-1 to Glucagon Production
Source: Int J Mol Sci. 2018 Nov 28;19(12):3791. doi: 10.3390/ijms19123791 (PMC6321596; doi:10.3390/ijms19123791)
Supplement: Supplementary file 1 [file ijms-19-03791-s001.pdf]

SUPPLEMENTARY DATA

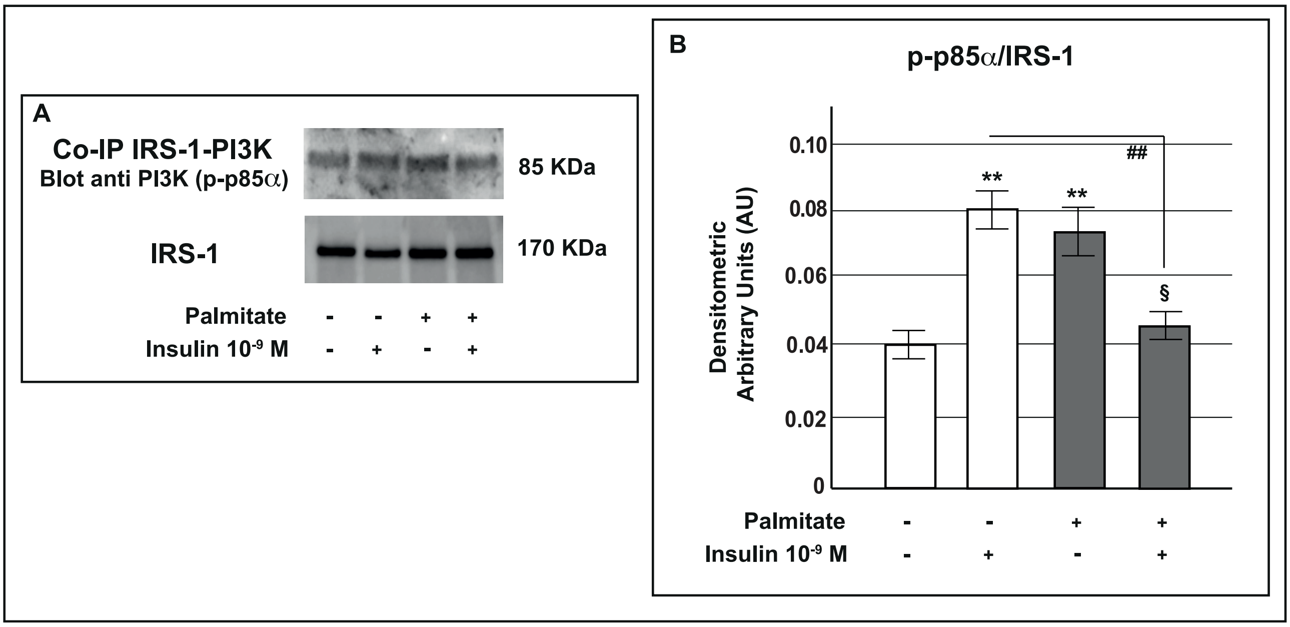

Supplementary Figure 1. Effect of pre-exposure to palmitate on PI3K activation in GLUTag cells.

Panel A: Representative immunoblot from control and palmitate GLUTag treated cells (0.5 mM for 24 h) acutely stimulated with insulin 10^{-9} M for 5 min for IRS-1-PI3K co-immunoprecipitation (p85α) and total IRS-1 (IRS-1); Panel B: corresponding densitometric analysis in control cells (open bars) and in cells exposed to palmitate (gray bars). ** $P < 0.01$, vs. basal control; ## $P < 0.01$ vs. insulin stimulated control group; §$P < 0.05$, vs. palmitate (1-way ANOVA followed by Bonferroni test, n = 3).

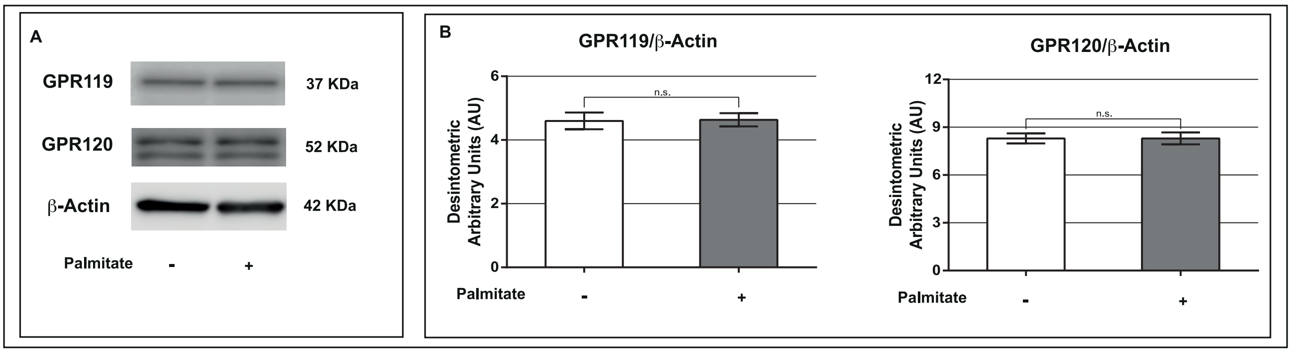

Supplementary Figure 2. Effect of pre-exposure to palmitate on GPR119 and GPR120 protein expression in GLUTag cells.

Panel A: Western blot analysis of GPR119 and GPR120 from palmitate GLUTag treated cells (0.5 mM for 24 h) with respect to control; Panel B: corresponding densitometric analysis. n.s.= not significant (1-way ANOVA followed by Bonferroni test, n = 3).

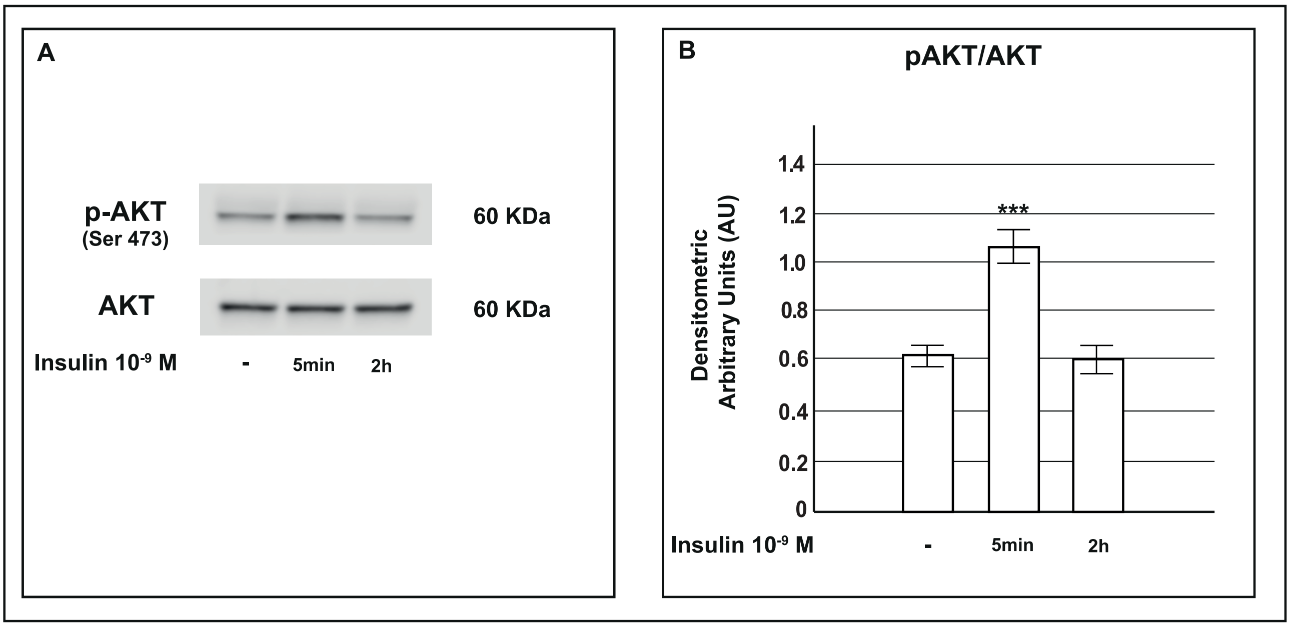

Supplementary Figure 3. Time course of p-AKT insulin-stimulation in GLUTag cells.

Panel A: Western blot analysis of p-AKT (Ser 473) and total AKT (AKT) from GLUTag cells stimulated with insulin 10-9 M for 5 min or 2h; Panel B: corresponding densitometric analysis. ***P < 0.001 vs. basal (1-way ANOVA followed by Bonferroni test, n = 3).
